# Endogenous Procoagulant Activity in Trauma Patients and Its Relationship to Trauma Severity

**DOI:** 10.1055/s-0038-1677030

**Published:** 2019-01-07

**Authors:** Shannon M. Prior, Myung S. Park, Kenneth G. Mann, Saulius Butenas

**Affiliations:** 1 Department of Biochemistry, University of Vermont, Colchester, Vermont, United States; 2 Division of Trauma, Critical Care, and General Surgery, Mayo Clinic, Rochester, Minnesota, United States

**Keywords:** factor XIa activity, tissue factor activity, severity of trauma, markers of trauma

## Abstract

**Background** It has been observed that trauma patients have elevated plasma procoagulant activity that could be assigned to an elevated concentration of tissue factor (TF). However, in many instances there is a discrepancy between the levels of TF and the procoagulant activity observed. We hypothesized that factor XIa (FXIa) could be responsible for this additional activity and that the presence and levels of both proteins could correlate with trauma severity.

**Methods** Citrate plasma from 98 trauma patients (47 blunt, 17 penetrating, and 34 thermal) were evaluated in clotting assays for the presence of FXIa and TF activity using respective inhibitory antibodies.

**Results** When the three trauma patient groups were divided into two cohorts (Injury Severity Score [ISS] > 25 and ISS ≤ 25), higher frequencies and concentrations of both TF and FXIa were observed for all the more severe injury subgroups.

**Conclusions** The majority of trauma patients have active FXIa in their plasma, with a significant fraction having active TF as well. Additionally, both TF and FXIa frequency and concentration directly relate to trauma severity. These data suggest the use of these two proteins as potential markers for the stratification of trauma patients.

## Introduction

Worldwide, trauma is the leading cause of death and disability in individuals younger than 44 years old. 1 Hemorrhage accounts for 30 to 45% of all trauma deaths and is the second most common cause for early death, with only central nervous system injuries being more frequent. 2 3 The ability of injured individuals to form a solid platelet and fibrin clot at the site of injury is vital for the prevention of exsanguination and for subsequent survival. The transmembrane glycoprotein tissue factor (TF) is an initiator of blood coagulation process leading to thrombin generation and consequential clot formation. 4 In healthy individuals, levels of detectable TF, if present, do not exceed 20 fM 5 ; however, upon vascular injury occurring during mechanical trauma, subendothelial TF is exposed to blood flow. 6 Traumatic injury also induces activation of inflammation, leading to the release of cytokines, which stimulate TF expression/exposure by monocytic cells and an increase in circulating TF-bearing microparticles. 7 8

One of the physiologic substrates of thrombin during the blood coagulation process is factor XI (FXI); however, FXIa can also be generated by FXIIa upon exposure to artificial surfaces 9 or to polyphosphates released from dying cells and/or platelets. 10 11 12 13 Although there are numerous serine protease inhibitors (serpins) circulating in blood at relatively high concentrations, none of them can efficiently inhibit FXIa. 14 The lack of such inhibitors suggests that when FXIa is formed, it can circulate in blood as an active enzyme for a substantial period of time.

Indeed, we observed in a previous study that the majority of trauma patients have both TF and FXIa present in plasma. 15 Similar results related to FXIa and TF activity were also obtained for patients with inflammatory bowel disease 16 and various cardiovascular diseases, 17 18 19 especially those with acute coronary syndrome, which have active FXIa in their plasma for which the concentrations correlates with markers of inflammation and thrombin generation. 17 A significant fraction of those patients also had detectable TF activity, which, most likely, could be assigned to TF presented on microparticles. It was established in such studies that these two proteins, TF and FXIa, are the only proteins present in patient plasma capable of initiating clot formation.

Results from previous studies along with the exposure of TF to blood flow in trauma patients, as well as the inflammatory response to injury, suggest that both FXIa and active TF could be present in plasma from trauma patients and may correlate with injury severity. In the current study, our objective was to examine FXIa and active TF from groups of patients with blunt, penetrating, and thermal injuries with the hypothesis that, in response to injury, patients with greater injury will have greater FXIa and active TF in their blood.

## Methods

### Study Design

This study was reviewed and approved by the Institutional Review Board (IRB H-03–014) at the Brooke Army Medical Center (BAMC). All severely burned and injured trauma patients with or without inhalation injury admitted to the intensive care unit (ICU) were considered for enrollment. Because the study was considered to be less than minimal risk by IRB, no consents were required. Inclusion criteria were: patients ≥18 years of age admitted within 24 hours from injury and an anticipated stay of 72 hours or greater at the U.S. Army Institute of Surgical Research (USAISR) Burn Center or Trauma ICU at BAMC. Patients and their families were given an information sheet concerning this less than minimal risk study prior to their enrollment. The following patients were excluded from the study: injury greater than 24 hours, prisoners, patients admitted to the wards, showing clinical signs of active hemorrhage, died within 72 hours after injury, patients undergoing therapeutic anticoagulation including Coumadin or antiplatelet agents, and those with known underlying coagulopathy. In addition, eight healthy volunteers were recruited and used as normal controls.

### Blood Sample Collection and Citrate Plasma Preparation

Baseline blood samples were collected into citrate tubes (9:1, 3.2% citrate) within 24 hours of admission (define as “Day 0”), followed by blood draws at 1, 2, 3, 5, and 7 days after admission. All samples were taken from an arterial or venous line inserted for standard clinical care. At the time of sample collection, no heparin was used in either arterial or venous lines due to the risk of heparin-induced thrombocytopenia. All the processing of blood samples was performed by trained research coordinators, at the clinical research laboratory of USAISR, which is located in the same facility as the Brooke Army Medical Center. Citrate blood-containing tubes were centrifuged immediately at 3,000 g for 15 minutes at room temperature and citrate plasma was frozen and stored at −70°C until assayed.

### Plasma Clotting Assays

These assays were done as previously described. 17 Citrate plasma was thawed at 37°C in the presence of corn trypsin inhibitor (prepared as previously described 20 ; prevents contact pathway initiation of coagulation). CaCl _2_ was added to a final 15 mmol/L concentration and the plasma incubated for 1 minute; clotting was initiated by the addition of 2 μmol/L phospholipid vesicles (PCPS) composed of 25% dioleoyl- *sn* -glycero-3-phospho-L-serine and 75% of 1,2-dioleoyl- *sn* -glycero-3-phosphocholine (both from Avanti Polar Lipids, Inc; Alabaster, Alabama, United States) and prepared as described previously. 21 In parallel, inhibitory monoclonal anti-FXI (αFXI-2) or anti-TF (αTF-5) antibodies (both produced in house) at a final 0.1 mg/mL concentration were individually added to the same plasma prior to the CaCl _2_ addition. αFXI-2 is specific for FXI/XIa and inhibits FIX activation by FXIa. 22 αTF-5 binds specifically to TF and interferes with the TF/FVIIa complex formation. 23 Clotting times were determined using the ST8 instrument (Diagnostica Stago, Parsippany, New Jersey, United States). FXIa and TF activity in plasma was calculated from calibration curves developed with human FXIa (a gift from Dr. R. Jenny from Haematologic Technologies, Inc., Essex Junction, Vermont, United States) or relipidated 20 TF _1–242_ (a gift from Dr. R. Lundblad from Baxter Healthcare Corp., Duarte, California, United States) in pooled 10-donor normal plasma.

### Statistical Analysis

Categorical demographics and presence of TF or FXI at baseline were reported as frequency (%) and compared across trauma types using the chi-square test. Continuous demographic variables were reported as median (interquartile range [IQR]), and raw FXIa levels were reported as 90th percentile (75th–100th). All comparisons of continuous variables across trauma types were made using the Wilcoxon rank-sum test. All *p* -values less than 0.05 were considered statistically significant.

## Results

### Patient Characteristics

From April 1, 2004, through May 31, 2005, a total of 479 burned and 1,366 nonburn trauma patients presented to the emergency department, and were screened for enrollment into study. Of these, 579 (212 burned and 367 nonburn trauma) patients were admitted to the ICU and blood samples obtained at specified time points. Three patients were subsequently excluded because they died within 48 hours after injury. Of these patients, 98 trauma patients had their plasma analyzed for TF and FXI activities. They were chosen based on mechanism of injury, divided into three groups based on the type of injury (thermal, blunt, or penetrating). Groups were comprised of 34 thermal patients (9 with inhalation injury), 47 blunt injury patients, and 17 penetrating injury patients ( Table 1 ). Overall, 44 patients (52%) had an Injury Severity Score (ISS) greater than 25. The median age of patients was 44.5 years and 80 of 98 patients (82%) were male. The median hospital stay for the entire study population was 19.5 days and a median of 11 days were spent in the ICU. Thirty-five blunt injury patients (79%), 7 penetrating injury (41%) patients, and 2 thermal injury patients (6%) had injuries to the head. All 98 patients were screened for hemorrhage at the time of blood draws; none were actively bleeding. For the majority of patient samples, prothrombin time (PT), activated partial thromboplastin time (aPTT), and fibrinogen levels were evaluated. Blunt patient samples ( *n* = 212) had an average PT value of 10.7 seconds (range, 9.3–30.2 seconds), an average aPTT value of 29.3 seconds (range, 15.5–75.1 seconds), and an average fibrinogen value of 571.9 mg/dL (range, 122.4–1,255.2 mg/dL). Penetrating patient samples ( *n* = 61) had an average PT value of 10.8 seconds (range, 9.4–25 seconds), an average aPTT value of 33.5 seconds (range, 17.0–78.1 seconds), and an average fibrinogen value of 560.4 mg/dL (range, 172.6–1,266.4 mg/dL). Thermal patient samples ( *n* = 174) had an average PT value of 12.3 seconds (range, 9.3–33.7 seconds), an average aPTT value of 36.3 seconds (range, 23.0– 17.3 seconds), and an average fibrinogen value of 532.1 mg/dL (range, 80.1–1,294.7 mg/dL). The eight healthy donors enrolled had an average PT value of 10.4 seconds (range, 9.5–11.4 seconds), an average aPTT value of 28.3 seconds (range, 24.1–32.2 seconds), and an average fibrinogen value of 307.7 mg/dL (range, 217.0–472.7 mg/dL). Eleven patients with thermal (34%), 5 with blunt (11%), and 1 with penetrating (6%) trauma (total 17) died during hospitalization. Seven of 11 thermal patients with a lethal outcome had third degree burn for >10% of their body surface and 5 of 11 had inhalation injuries. Four of five blunt trauma patients who died had head–neck injuries (three patients with Abbreviated Injury Scale [AIS] = 5 and one with AIS = 3). The death of the single patient with a penetrating trauma was caused by a chest–thorax injury (AIS = 5). For patients with a lethal outcome, the mean survival time after admission was 23 ± 38 days (range, 2–126 days).

**Table 1:** Patient characteristics

Injury	Age	Men	ISS	ISS > 25	Hospital days a	ICU days	In-hospital mortality	Injury to head
Blunt ( *n* = 47)	40 (29, 59)	37 (79%)	27 (17, 34)	28 (60%)	20.5 (14, 32)	12 (6, 20)	5 (11%)	35 (75%)
Penetrating ( *n* = 17)	33 (25, 48)	16 (94%)	18 (16, 26)	6 (36%)	15.5 (11, 26.5)	5 (3, 11)	1 (6%)	7 (41%)
Thermal ( *n* = 34)	49 (34, 70)	27 (79%)	25 (16, 26)	10 (29%)	50 (22, 62)	14.5 (5, 44)	11 (34%)	2 (6%)
Overall ( *n* = 98)	44.5 (29, 61)	80 (82%)	25 (17, 34)	44 (45%)	19.5 (12, 39)	11 (5, 21)	17 (17%)	44 (45%)
*p* -Value	0.033	0.34	0.035	0.018	<0.001	0.008	0.015	<0.001

a In-hospital mortalities excluded.

Statistical analysis was performed across trauma groups for all demographic parameters presented in Table 1 . Sex was the only parameter that was not statistically significant, whereas age, ISS, ISS > 25, and in-hospital mortality are reported as significant with *p* -values between 0.05 and 0.01 and hospital days and injury to head reported as significant with *p* -values < 0.01.

### Tissue Factor Activity

Overall, 468 samples from 98 patients were processed for TF activity ( Table 2 ), with 30% of baseline samples and 16.3% of samples overall (53% of patients) containing active TF. Eight healthy controls were also analyzed with none having detectable TF activity. The mean TF concentration from the 98 baseline samples was 0.32 ± 0.52 pM, whereas across all samples the mean TF concentration was 0.18 ± 0.42 pM.

**Table 2:** Frequencies and concentrations of TF in plasma samples from patients within each trauma category along with all samples combined

TF	Blunt		Penetrating		Thermal		All trauma types	
	Day 0 ( *n* = 47)	Overall ( *n* = 219)	Day 0 ( *n* = 17)	Overall ( *n* = 63)	Day 0 ( *n* = 34)	Overall ( *n* = 186)	Day 0 ( *n* = 98)	Overall ( *n* = 468)
Frequency	26%	17%	29%	21%	35%	15%	30%	16%
Mean (pM)	0.26 ± 0.44	0.18 ± 0.41	0.43 ± 0.76	0.27 ± 0.57	0.35 ± 0.49	0.15 ± 0.36	0.32 ± 0.52	0.18 ± 0.42
Range (pM)	0 to <1	0–2.3	0–2.3	0–2.3	0 to <1	0–1.4	0–2.3	0–2.3
Frequency of patients	12 of 47 (26%)	24 of 47 (51%)	5 of 17 (29%)	10 of 17 (59%)	12 of 34 (35%)	18 of 34 (53%)	29 of 98 (30%)	52 of 98 (53%)

**Fig. 1:**
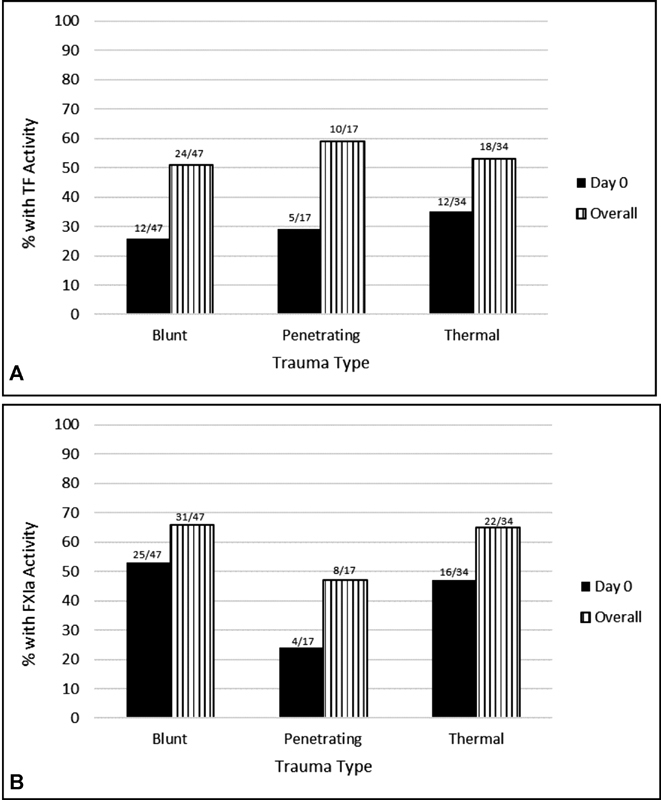
TF and FXIa activity. Frequency of functional tissue factor (TF) **(A)** and active factor XIa (FXIa) **(B)** in plasma from trauma patients at Day 0 and during the entire observation period of 7 days.

Blunt injury patient samples comprised 48% of baseline samples and 47% of the total number of samples analyzed. From the blunt injury baseline samples, 26% had active TF resulting in a mean of 0.26 ± 0.44 pM ( Fig. 1 ). TF frequency decreased with time, and for all blunt patient samples, the frequency of active TF dropped to 17% (51% of blunt patients) resulting in a mean concentration of 0.18 ± 0.41 pM.

The smallest number of patients and corresponding samples was in the group of penetrating injury patients, which made up 17% of total baseline samples and 14% of samples overall. At baseline, 29% of penetrating injury samples had active TF resulting in a mean of 0.43 ± 0.27 pM. Similar to blunt injury patients, the frequency of TF in penetrating injury samples decreased with time from 29% in baseline samples to 21% overall (59% of penetrating patients) resulting in a mean of 0.27 ± 0.57 pM.

Thermal injury samples comprised a similar number of baseline and overall samples as blunt injury samples (35 and 40%, respectively). Thirty-five percent of baseline samples taken from thermal injury patients had active TF resulting in a mean concentration of 0.35 ± 0.49 pM, while, similarly to the other two groups, only 15% of samples overall (53% of thermal patients) had active TF resulting in a mean concentration of 0.15 ± 0.36 pM. Of the nine thermal patients presenting with inhalation injury, five patients had TF present at baseline resulting in a mean concentration of 0.56 ± 0.53 pM, while overall the mean concentration was 0.2 ± 0.44 pM.

When the frequency of TF at baseline was compared across blunt, penetrating, and thermal injury trauma groups (26, 29, and 35%, respectively), a *p* -value of 0.64 was obtained, indicating no statistical significance. Statistical analysis was not performed for the frequency of TF across all samples as patients had varying numbers of observations, nor was it performed for TF concentrations given that the majority of samples with TF present were equal to 1 pM and it could not have been treated as a continuous variable.

### Factor XIa Activity

Of the 98 baseline samples taken upon Day 0, 46% had detectable FXIa with a mean concentration of 19.6 ± 32.2 pM, whereas only 23% of the total 468 samples collected had detectable levels of FXIa (62% of all patients) with a mean concentration of 9.0 ± 22.5 pM ( Table 3 ). None of the eight healthy controls analyzed had detectable FXIa activity.

**Table 3:** Frequencies and concentrations of FXIa in plasma samples from patients within each trauma category along with all samples combined

FXIa	Blunt		Penetrating		Thermal		All trauma types	
	Day 0 ( *n* = 47)	Overall ( *n* = 219)	Day 0 ( *n* = 17)	Overall ( *n* = 63)	Day 0 ( *n* = 34)	Overall ( *n* = 186)	Day 0 ( *n* = 98)	Overall ( *n* = 468)
Frequency	53%	29%	24%	19%	47%	20%	46%	23%
Mean (pM)	20.3 ± 27.3	10.3 ± 23.0	19.2 ± 48.1	10.6 ± 30.9	18.8 ± 29.9	6.8 ± 18.1	19.6 ± 32.2	9.0 ± 22.5
Range (pM)	0–112	0–162	0–160	0–160	0–134	0–134	0–160	0–162
Frequency of patients	25 of 47 (53%)	31 of 47 (66%)	4 of 17 (24%)	8 of 17 (47%)	16 of 34 (47%)	22 of 34 (65%)	45 of 98 (46%)	61 of 98 (62%)

For blunt injury patient baseline samples, 53% had detectable FXIa resulting in a mean concentration of 20.3 ± 27.3 pM. Similar to TF, the frequency of FXIa decreased with time, and for all samples analyzed from blunt trauma patients, 219 of total 468 samples (29%) had detectable FXIa (66% of blunt patients) resulting in a mean concentration of 10.3 ± 23.0 pM.

The smallest patient group was those who had penetrating injuries; 24% of ICU Day 0 samples contained detectable FXIa resulting in a mean of 19.2 ± 48.1 pM, whereas of all penetrating trauma samples (63 of total 468 samples) 19% had detectable levels of FXIa (47% of penetrating patients), resulting in a mean of 10.6 ± 30.9 pM.

For patients with thermal trauma, 47% of Day 0 samples contained detectable FXIa, resulting in a mean of 18.8 ± 29.9 pM. For all thermal trauma samples (186 of total 468 samples), 20% had detectable FXIa (65% of thermal patients) resulting in a mean of 6.8 ± 18.1 pM. Of the nine thermal patients presenting with inhalation injury, six patients had active FXIa present at baseline, resulting in a mean concentration of 29.7 ± 28.0 pM, while overall the mean concentration was 11.4 ± 21.6 pM.

When the frequency of FXIa at baseline was compared across blunt, penetrating, and thermal injury trauma groups (53, 24, and 47%, respectively), a *p* -value of 0.11 was obtained, indicating no statistical significance. Similarly, when the 90th percentile of FXIa concentrations at baseline was compared across blunt, penetrating, and thermal injury trauma groups (60, 130, and 55 pM, respectively), a *p* -value of 0.21 was obtained, indicating no statistical significance. Statistical analysis was not performed for the frequency of FXIa across all samples as patients had varying numbers of observations, nor was it performed for all FXIa concentrations given that so many samples had a value of 0 pM.

### TF and FXIa versus ISS

**Fig. 2:**
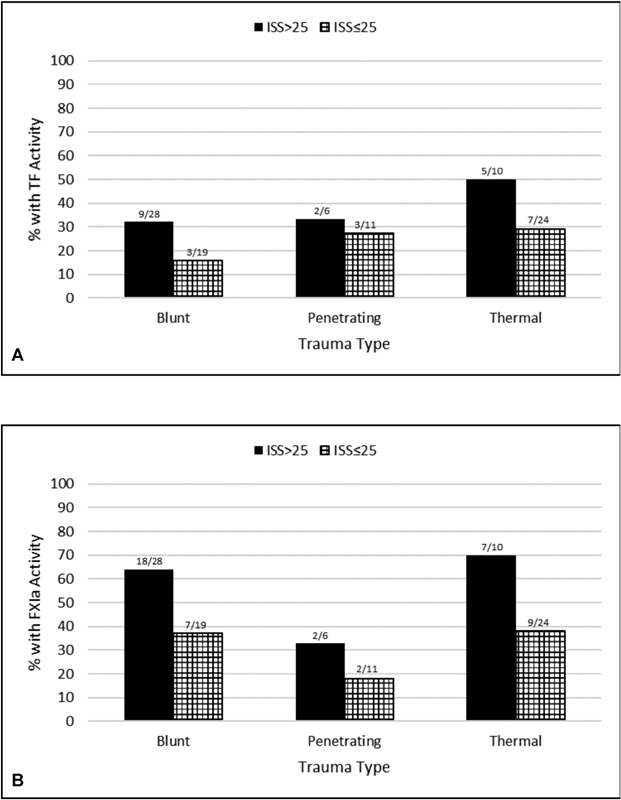
TF and FXIa frequency versus ISS. Frequency of functional tissue factor (TF) **(A)** and active factor XIa (FXIa) **(B)** in plasma from trauma patients at Day 0 for subgroups with an Injury Severity Score (ISS) > 25 and ISS ≤25.

Each group of trauma patients, blunt ( *n* = 47), penetrating ( *n* = 17), and thermal ( *n* = 34), was split into two subgroups based on those with ISS > 25 and those with ISS ≤ 25. For these subgroups, only baseline samples were analyzed for the frequencies of TF and FXIa and mean values ( Fig. 2 ).

**Fig. 3:**
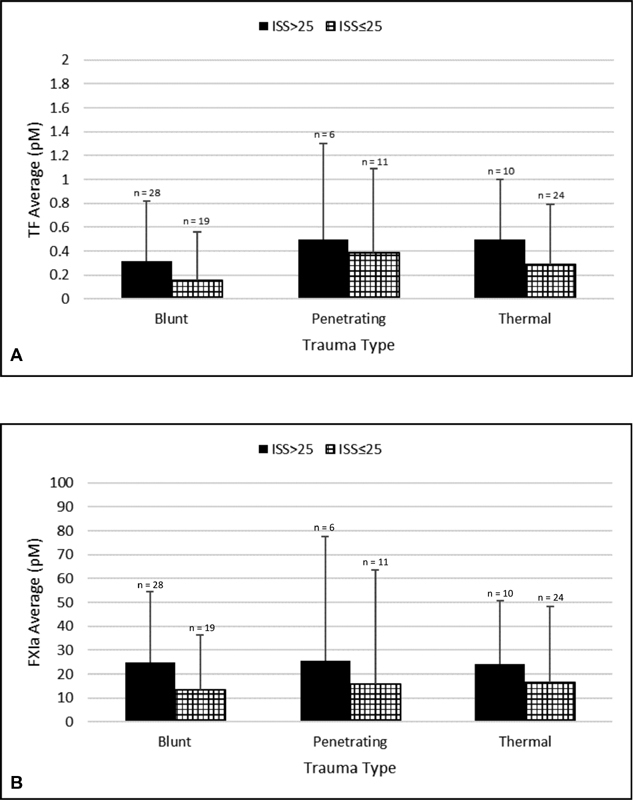
TF and FXIa levels versus ISS. Mean values of functional tissue factor (TF) **(A)** and active factor XIa (FXIa) **(B)** in plasma from trauma patients at Day 0 for subgroups with an Injury Severity Score (ISS) > 25 and ISS ≤25.

The most pronounced difference in the frequency of TF in subgroups ISS ≤ 25 and ISS > 25 was observed for blunt trauma patients, with a twofold higher prevalence in the ISS > 25 subgroup (15.8 and 32.1%, respectively). Similarly, a 1.8-fold higher prevalence of FXIa was observed for the blunt patient subgroup ISS > 25 (36.8 vs. 64.3%), with frequencies higher than those observed for TF. In regard to TF concentrations, the same pattern was observed with a twofold higher concentration in the ISS > 25 blunt patient subgroup (0.16 ± 0.4 vs. 0.32 ± 0.5 pM). Additionally, the same pattern was observed for FXIa in which concentrations were 1.8-fold higher in the blunt patient subgroup ISS > 25 (13.5 ± 22.8 vs. 24.9 ± 29.5 pM) ( Fig. 3 ).

In contrast to blunt patients, the least pronounced difference in TF frequency in subgroups ISS ≤ 25 and ISS > 25 was observed for penetrating patients (27.3 and 33.0%, respectively). FXIa frequency in penetrating patients was at a lower frequency than that in blunt patients, although the 1.8-fold difference in subgroups ISS ≤ 25 and ISS > 25 remained consistent (18.2 and 33.0%, respectively). For TF concentrations in the penetrating patient subgroups ISS ≤ 25 and ISS > 25, there was only a 1.3-fold increase as opposed to the twofold increase observed for blunt patients, although slightly higher mean values were obtained (0.4 ± 0.7 and 0.5 ± 0.8 pM, respectively). A slightly lower increase (1.6-fold) was also observed for FXIa concentrations in ISS ≤ 25 and ISS > 25 subgroups of penetrating patients versus blunt patients, although with similar concentrations (15.7 ± 48 and 25.5 ± 52 pM, respectively).

Thermal patients had the highest frequencies of both TF and FXIa in either subgroup. For TF in subgroups ISS ≤ 25 and ISS > 25, there was a 1.7-fold increase in frequencies (29.2 and 50%, respectively) and also a 1.7-fold increase in mean values (0.29 ± 0.5 and 0.5 ± 0.5 pM, respectively). For FXIa in subgroups ISS ≤ 25 and ISS > 25, a 1.9-fold increase in frequencies was observed (37.5 and 70%, respectively) along with a 1.5-fold increase in mean values (16.6 ± 31.5 and 24.1 ± 26.5 pM, respectively).

## Discussion

This study evaluates the prevalence and levels of TF and FXIa within 24 hours of injury and during the course of the hospital stay for groups of blunt, penetrating, and thermal injury trauma patients. Although thermal injury patients had the highest Day 0 frequency of TF, penetrating injury patients maintained the highest overall frequency along with the highest TF levels in both Day 0 samples and in all samples analyzed.

When all three trauma groups were subdivided into ISS ≤ and ISS > 25, there was a twofold higher TF frequency and concentration in the higher ISS subgroup for both blunt and thermal trauma patients. In contrast, frequency and concentration were only 1.2- and 1.3-fold higher, respectively, in the penetrating trauma subgroup of ISS > 25 as compared with ISS ≤ 25. It is perhaps expected that this group of penetrating injury patients would have less of a correlation between TF and ISS due to sample size, as they comprised the smallest of all groups ( *n* = 17) with less than half having ISS > 25, and the fact that they had the lowest mean ISS score of all. The lack of a distinguishable and significant pattern of TF prevalence and/or concentration over time is consistent with a previous study 24 and would require a larger sample size and mean hospital stay to be conclusive.

In this study, we observed that the highest frequency of FXIa in both initial (within 24 hours) samples and also during the entire hospital stay was in the group of blunt injury patients, whereas the lowest frequencies in both sample sets were observed in penetrating patients. In terms of mean FXIa levels, however, there was no pronounced difference in all three groups. The elevated frequency of FXIa in blunt patients compared with thermal and, even more pronounced, to penetrating patients may be explained by injury mechanism and the subsequent elevation of apoptosis. The most common causes of blunt trauma injuries are due to motor vehicle collisions, falls, industrial/recreational accidents, etc., in which a large number of patients suffer from traumatic brain injury and/or pulmonary injury—both of which are shown to lead to increased apoptosis and the subsequent release of histones and polyphosphates into the extracellular space. 25 26 As discussed earlier, FXIa can be generated by FXIIa upon exposure to organic polyphosphates 10 11 and also histones, which enhance polyphosphate-driven thrombin generation, 27 released from dying cells. This hypothesis is supported by the observation that in this study, blunt injury patients had the highest rate of head injuries as compared with penetrating and thermal injury patients. Although thermal injury patients had the lowest rate of head injuries, it should be noted that burns themselves are known to lead to increased apoptotic cell death, 28 29 which may explain why the frequency of FXIa is more similar in blunt and thermal patients versus penetrating patients.

When each group of trauma patients was divided into the subgroups ISS > and ISS ≤ 25, there was a similar higher prevalence of FXIa in high ISS subgroups across all three injury groups. That is in contrast to that observed for TF, in which penetrating patients responded less to severe versus nonsevere injury compared with blunt and thermal patients. Similar to frequency, each group had higher levels of FXIa for subgroups of ISS > 25 although no pronounced difference was observed in the increase across blunt, penetrating, and thermal patient subgroups.

There are several limitations in this study. (1) Analysis was focused on TF and FXIa and we did not simultaneously assess other potential biomarkers that could provide further insight to trauma-induced coagulopathy (TIC). However, we have already published our observations of derangements in whole blood (thromboelastography [TEG]) and plasma (PT, PTT, antithrombin III [ATIII], protein C activity). 30 We found that, although standard coagulation markers are the same after injury, TEG, activated protein C, and ATIII were consistent with a hypercoagulable profile. The purpose of this paper is to focus on TF and FXIa activities, to see if their occurrence correlates with trauma severity. (2) Due to overall small number of patients, we did not distinguish burn patients into two separate categories (those with or without inhalation injury). (3) ISS is not entirely reflective of injury severity in thermal patients; however, the goal of this paper was to compare levels of TF and FXIa in three different groups of trauma patients separated by severe versus nonsevere injury, which was defined by the same criteria across all groups (ISS). For the definition of severe injury, ISS > 25 was chosen as it was the threshold in the previously published study using thrombin generation. 30 (4) Blood samples were obtained from either arterial or venous line, and this could have biased the results. However, the standard clinical procedural guideline was followed in the management of lines and collection of blood samples from these lines. This entailed wasting 10 mL of blood from the line, ensuring that the sample was free of the various diluents from either line. Also, it was not practical to perform percutaneous blood draws when existing lines were available. The IRB has specifically requested that we minimize blood draws. (5) The patients whose samples were analyzed were not consecutive admissions but nonrandomly chosen based on their mechanism of injury. (6) Our major goal was to assess the role of two novel biomarkers in relation to injury severity. Future studies to characterize the profiles FXIa and active TF over time, taking into account shock, hypotension, and transfusions, would enhance our understanding of their role in the TIC after injury. To assess whether these novel assays could find practical applications as diagnostic indicators or be predictive of TIC, we wanted to establish that their values varied in response to injury severity. Verification of the presence of such variability is essential before a larger and more costly study is embarked.

In conclusion, this study demonstrates that FXIa and active TF are quantifiable in the plasma derived from blunt, penetrating, and thermal injury trauma patients; their concentrations relate to injury severity. FXIa and active TF could therefore serve as potential new biomarkers of trauma severity and detection of these proteins may ultimately assist in the improved treatment for trauma patients. Future studies to characterize the profiles FXIa and active TF over time, taking into account shock, hypotension, and transfusions, would enhance our understanding of their role in TIC after injury and may lead to improved assays that are more applicable for use in the clinical setting. In short, it remains to be determined if the characterization of these biomarkers could find practical applications.

## References

[1] GBD 2013 Mortality and Causes of Death Collaborators.Global, regional, and national age-sex specific all-cause and cause-specific mortality for 240 causes of death, 1990-2013: a systematic analysis for the Global Burden of Disease Study 2013 Lancet 2015 385(9963):117 171 25530442 10.1016/S0140-6736(14)61682-2 PMC4340604

[2] Kauvar D S Lefering R Wade C E Impact of hemorrhage on trauma outcome: an overview of epidemiology, clinical presentations, and therapeutic considerations J Trauma 2006 60(6, Suppl):S3 S11 16763478 10.1097/01.ta.0000199961.02677.19

[3] Sauaia A Moore F A Moore E E Epidemiology of trauma deaths: a reassessment J Trauma 1995 38 02 185 193 7869433 10.1097/00005373-199502000-00006

[4] Butenas S Tissue factor structure and function Scientifica (Cairo) 2012 2012 964862 24278763 10.6064/2012/964862 PMC3820524

[5] Butenas S Bouchard B A Brummel-Ziedins K E Parhami-Seren B Mann K G Tissue factor activity in whole blood Blood 2005 105 07 2764 2770 15604222 10.1182/blood-2004-09-3567

[6] Wang P Ba Z F Chaudry I H Endothelial cell dysfunction occurs very early following trauma-hemorrhage and persists despite fluid resuscitation Am J Physiol 1993 265(3 Pt 2):H973 H979 8214134 10.1152/ajpheart.1993.265.3.H973

[7] Gando S Kameue T Nanzaki S Hayakawa T Nakanishi Y Participation of tissue factor and thrombin in posttraumatic systemic inflammatory syndrome Crit Care Med 1997 25 11 1820 1826 9366764 10.1097/00003246-199711000-00019

[8] Utter G H Owings J T Jacoby R C Gosselin R C Paglieroni T G Injury induces increased monocyte expression of tissue factor: factors associated with head injury attenuate the injury-related monocyte expression of tissue factor J Trauma 2002 52 06 1071 1077, discussion 1077 12045632 10.1097/00005373-200206000-00008

[9] MacFarlane R G An enzyme cascade in the blood clotting mechanism, and its function as a biochemical amplifier Nature 1964 202 498 499 14167839 10.1038/202498a0

[10] Gansler J Jaax M Leiting S Structural requirements for the procoagulant activity of nucleic acids PLoS One 2012 7 11 e50399 23226277 10.1371/journal.pone.0050399 PMC3511531

[11] Kannemeier C Shibamiya A Nakazawa F Extracellular RNA constitutes a natural procoagulant cofactor in blood coagulation Proc Natl Acad Sci U S A 2007 104 15 6388 6393 17405864 10.1073/pnas.0608647104 PMC1851071

[12] Müller F Mutch N J Schenk W A Platelet polyphosphates are proinflammatory and procoagulant mediators in vivo Cell 2009 139 06 1143 1156 20005807 10.1016/j.cell.2009.11.001 PMC2796262

[13] Smith S A Morrissey J H Polyphosphate: a new player in the field of hemostasis Curr Opin Hematol 2014 21 05 388 394 25010799 10.1097/MOH.0000000000000069 PMC4415375

[14] Wuillemin W A Minnema M Meijers J C Inactivation of factor XIa in human plasma assessed by measuring factor XIa-protease inhibitor complexes: major role for C1-inhibitor Blood 1995 85 06 1517 1526 7534133

[15] Prior S M Cohen M J Conroy A S Correlation between factor (F)XIa, FIXa and tissue factor and trauma severity J Trauma Acute Care Surg 2017 82 06 1073 1079 28328676 10.1097/TA.0000000000001449 PMC5436934

[16] Undas A Owczarek D Gissel M Salapa K Mann K G Butenas S Activated factor XI and tissue factor in inflammatory bowel disease Inflamm Bowel Dis 2010 16 09 1447 1448 20049947 10.1002/ibd.21206 PMC3152204

[17] Butenas S Undas A Gissel M T Szuldrzynski K Zmudka K Mann K G Factor XIa and tissue factor activity in patients with coronary artery disease Thromb Haemost 2008 99 01 142 149 18217146 10.1160/TH07-08-0499

[18] Undas A Slowik A Gissel M Mann K G Butenas S Circulating activated factor XI and active tissue factor as predictors of worse prognosis in patients following ischemic cerebrovascular events Thromb Res 2011 128 05 e62 e66 21820158 10.1016/j.thromres.2011.06.010 PMC3205247

[19] Ząbczyk M Butenas S Plicner D Fijorek K Sadowski J Undas A Factors associated with the presence of circulating active tissue factor and activated factor XI in stable angina patients Blood Coagul Fibrinolysis 2012 23 03 189 194 22343680 10.1097/MBC.0b013e32834ee194

[20] Cawthern K M van 't Veer C Lock J B DiLorenzo M E Branda R F Mann K G Blood coagulation in hemophilia A and hemophilia C Blood 1998 91 12 4581 4592 9616154

[21] Higgins D L Mann K G The interaction of bovine factor V and factor V-derived peptides with phospholipid vesicles J Biol Chem 1983 258 10 6503 6508 6406482

[22] Butenas S Dee J D Mann K G The function of factor XI in tissue factor-initiated thrombin generation J Thromb Haemost 2003 1 10 2103 2111 14521591 10.1046/j.1538-7836.2003.00431.x

[23] Parhami-Seren B Butenas S Krudysz-Amblo J Mann K G Immunologic quantitation of tissue factors J Thromb Haemost 2006 4 08 1747 1755 16879217 10.1111/j.1538-7836.2006.02000.x

[24] Butenas S Manning C Freeman K Factor XIa, factor IXa and tissue factor activity in blunt trauma patients J Thromb Haemost 2015 13 02 569

[25] Hotchkiss R S Schmieg R E Jr Swanson P E Rapid onset of intestinal epithelial and lymphocyte apoptotic cell death in patients with trauma and shock Crit Care Med 2000 28 09 3207 3217 11008984 10.1097/00003246-200009000-00016

[26] Liener U C Knöferl M W Sträter J Induction of apoptosis following blunt chest trauma Shock 2003 20 06 511 516 14625474 10.1097/01.shk.0000095057.62263.fb

[27] Semeraro F Ammollo C T Morrissey J H Extracellular histones promote thrombin generation through platelet-dependent mechanisms: involvement of platelet TLR2 and TLR4 Blood 2011 118 07 1952 1961 21673343 10.1182/blood-2011-03-343061 PMC3158722

[28] Lightfoot E Jr Horton J W Maass D L White D J McFarland R D Lipsky P E Major burn trauma in rats promotes cardiac and gastrointestinal apoptosis Shock 1999 11 01 29 34 9921713 10.1097/00024382-199901000-00004

[29] Nakanishi T Nishi Y Sato E F Ishii M Hamada T Inoue M Thermal injury induces thymocyte apoptosis in the rat J Trauma 1998 44 01 143 148 9464763 10.1097/00005373-199801000-00019

[30] Park M S Xue A Spears G M Thrombin generation and procoagulant microparticle profiles after acute trauma: A prospective cohort study J Trauma Acute Care Surg 2015 79 05 726 731 26496097 10.1097/TA.0000000000000839 PMC4621757

